# Supplementary material for: Prevalence and predictors of secondary traumatic stress symptoms in health care professionals working with trauma victims: A cross-sectional study
Source: PLoS One. 2021 Feb 23;16(2):e0247596. doi: 10.1371/journal.pone.0247596 (PMC7901735; doi:10.1371/journal.pone.0247596)
Supplement: S1 Table — (DOC) [file pone.0247596.s001.doc]

STROBE Statement—Checklist of items that should be included in reports of ***cross-sectional studies***

|  | Item No | Recommendation |
| --- | --- | --- |
| **Title and abstract** | 1 | (*a*) Prevalence and predictors of secondary traumatic stress symptoms in health care professionals working with trauma victims: a cross-sectional study |
|  |  | (*b*) The manuscript provides the data about prevalence of secondary traumatic stress among professionals (nurses and paramedics) working with trauma victims and its relationship with work-related variables, i.e., occupational load, job satisfaction, social support and cognitive processing of trauma in the form of cognitive coping strategies. The findings show that these factors may play an important role in secondary traumatic stress symptoms. |
| Introduction | | |
| Background/rationale | 2 | Medical personnel is an occupational group that is especially prone to secondary traumatic stress. The factors conditioning its occurrence include organizational and work-related factors, as well as personal features and traits. The aim of this study was to determine Secondary Traumatic Stress (STS) indicators in a group of medical personnel, considering occupational characteristics, job satisfaction, social support, and cognitive processing of trauma. |
| Objectives | 3 | It was assumed that the main determinant of STS will be cognitive trauma processing and that negative strategies (regret, denial) will be positively linked with STS symptoms and positive strategies, especially resolution/acceptance and positive cognitive restructuring will be linked negatively. It was assumed in the study that STS symptoms will be positively connected with the occupational load indicators and negatively with job satisfaction and social support. |
| Methods | | |
| Study design | 4 | Cross-sectional study. |
| Setting | 5 | 430 representatives of medical personnel who provide medical help to trauma victims were included in the study. The study was conducted in the period from November 2019 to February 2020 in 12 units and included voivodship rescue stations, emergency medical teams, emergency wards in several Polish hospitals as well as cancer wards, intensive care units, and hospices. |
| Participants | 6 | The analysis included the results of 419 (11 questionnaires were dropped out due to the missing data; only participants with complete questionnaires were included in the analyses) people in the age from 19 to 65 years (M=39.60, SD=11.03) who completed questionnaires provided for them. The study inclusion criterion was performing the profession of a paramedic or nurse and work with people who had traumatic experiences. Among study participants, there were 137 (32.7%) men and 282 (67.3%) women. The studied group included paramedics (n=201) where 60.2% were men and nurse staff (n=218) in which there was a significant majority of women (92.7%). |
| Variables | 7 | Dependent variable: Secondary traumatic stress  Independent variables (predictors): occupational load (work experience, number of working hours per week with patients after traumatic experience, workload), social support (from supervisors, co-workers, family, friends), cognitive trauma processing in the form of cognitive coping strategies (positive cognitive restructuring, downward comparison, resolution/acceptance, denial, regret).  Confounders: Occupational groups (nurses, paramedics), age, gender. |
| Data sources/ measurement | 8* | Secondary Traumatic Stress Inventory  Job Satisfaction Scale  Social Support Scale – What Support Can You Expect  Cognitive Processing of Trauma Scale (CPOTS) |
| Bias | 9 | The validated measures, criteria of study inclusion, objective data source, was used to avoid bias. |
| Study size | 10 | 430 representatives of medical personnel who provide medical help to trauma victims were included in the study. The analysis included the results of 419 (11 questionnaires were dropped out due to the missing data; only participants with complete questionnaires were included in the analyses). The study was conducted in the period from November 2019 to February 2020 in 12 units and included voivodship rescue stations, emergency medical teams, emergency wards in several Polish hospitals as well as cancer wards, intensive care units, and hospices. The authors collected the data from the participants during the work shifts. |
| Quantitative variables | 11 | Gender (male, female) and occupational group (nurses, paramedics); t-Student’s test was used for analysis. |
| Statistical methods | 12 | The IBM SPSS, version 25 software was used to verify the obtained data. The two-tailed probability value of < 0.05 was considered to be statistically significant. The first step of data analysis consisted of the calculation of descriptive statistics that included mean and standard deviation for secondary traumatic stress, occupational characteristic, job satisfaction, social support and cognitive coping strategies. Additional for demographic characteristic frequency and percentage were computed. T-Student’s test was implemented to compare the differences in the prevalence of secondary traumatic stress between nurses and paramedics as well as between men and women. Pearson’s correlation coefficients were applied to analyze the relations between the variables. The Benjamini-Hochberg procedure was done for multiple comparisons. A multivariable stepwise regression analysis was used in order to find a dependent variable predictor (STS) among independent variables (occupational load characteristic, job satisfaction, cognitive trauma processing, and social support). To assess model fit, R^2^ was used. Moreover, regression analysis provided data that include adjusted R^2^, R^2^-changes, standardized regression coefficient (β), unstandardized regression coefficient (B), F-statistic, confident intervals for B, and *p*-value. Multicollinearity was checked by using tolerance (> 0.10) and variance inflation factor (< 5). |
|  |  |  |
|  |  |  |
|  |  |  |
|  |  |  |
| Results | | |
| Participants | 13* | The analysis included the results of 419 (11 questionnaires were dropped out due to the missing data; only participants with complete questionnaires were included in the analyses). |
| Descriptive data | 14* | The analysis included the results of 419 (11 questionnaires were dropped out due to the missing data; only participants with complete questionnaires were included in the analyses) people in the age from 19 to 65 years (M=39.60, SD=11.03) who completed questionnaires provided for them. The study inclusion criterion was performing the profession of a paramedic or nurse and work with people who had traumatic experiences. Among study participants, there were 137 (32.7%) men and 282 (67.3%) women. The studied group included paramedics (n=201) where 60.2% were men and nurse staff (n=218) in which there was a significant majority of women (92.7%). The majority of paramedics provide help for people who experienced various accidents, especially road accidents (57.2%) but also after traumas like strokes and cardiac infarction (42.8%). Nurse staff included people working with cancer patients (87.7%) and accident sufferers (18.3%). Work experience of the medical personnel who participated in the study amounted at from 1 year to 43 years (M=12.18, SD=9.75), the number of work hours devoted to helping injured patients amounted at 2 to 90 (M=38.24, SD=15.64), and workload expressed by a percent of work devoted directly to providing help to patients in relation to the whole performed work from 2 to 100% (M=69.11, SD=31.89). |
| Outcome data | 15* | *see: Table 1 in the manuscript |
| Main results | 16 | Paramedics and nurses are significantly exposed to the occurrence of secondary traumatic stress symptoms. Considering 33 points assumed as a cut-off point for the general STS result it was reported that 237 people which constitutes 56.6% of study participants show low or moderate STS symptom levels. In turn, high levels of these symptoms signifying a high probability of STSD diagnosis occurred in 182 people which is 43.4% of study participants. The nurse staff representatives symptoms (STS for nurses: M=32.23, SD=20.69, STS for paramedics: M=29.67, SD=18.28; t[417]=-1.336, p>0.05). Gender did not differentiate the level of STSD symptoms (men: M=30.32, SD=18.31; women: M=31.33, SD=20.20; t[417]=-0.496, *p* > 0.05). Positive links occur between the age of study participants and STS (r = 0.123, p<0.05).  Two of the three occupational load indicators found to be negatively related to STS number of workhours per week, workload). Job satisfaction, social support from supervisors, family and friends, and two positive cognitive coping strategies in the form of positive cognitive restructuring and resolution/acceptance was also negatively connected with STS. Regret and denial revealed positive associations with STS. Regression model explains 34% variance of STS. The main predictor of STS symptoms was job satisfaction. |
| Other analyses | 17 | - |
| Discussion | | |
| Key results | 18 | The results showed that the main predictor of STS symptoms in the studied group of medical personnel is job satisfaction. In the explanation of STS symptoms occurrence two cognitive strategies are also applied, that is regret (positive relation) and resolution/acceptance (negative relation). Occupational load and social support do not allow the prediction of negative consequences of secondary trauma exposure among the medical personnel. |
| Limitations | 19 | It was a cross-sectional study which does not allow to draw conclusions related to the cause and effect dependencies. Subjective indicators of indirect exposure to trauma e.g. in the form of evaluation of the size and meaning of the influence of the events experienced by patients treated as the severity of the perceived trauma were also not taken into account. The meaning of personal traumatic experiences that could influence the level of STS symptoms was also not analyzed. The analyses did not include a certain place of work (hospital, ward) because of the possible simultaneous employment of study participants in multiple places. It should be underlined that the study group was not homogeneous. Men constituted the majority of the group of paramedics while the group of nurses included mainly women. |
| Interpretation | 20 | The conducted research have a practical implications for the development of prevention programs aiming at the decrease of levels of STS symptoms and lowering the risk of STS occurrence. The job satisfaction through increasing its value and giving meaning to the performed action should also be taken into account. The development of competences of coping with trauma considering the alteration of cognitive coping strategies from negative to positive as well as encouragement to use of various self-care practices is also advisable. |
| Generalisability | 21 | Slightly reduced due to the lack of gender equality in individual occupational groups. |
| Other information | | |
| Funding | 22 | Internal grant of University of Opole, Poland. |

*Give information separately for exposed and unexposed groups.

**Note:** An Explanation and Elaboration article discusses each checklist item and gives methodological background and published examples of transparent reporting. The STROBE checklist is best used in conjunction with this article (freely available on the Web sites of PLoS Medicine at http://www.plosmedicine.org/, Annals of Internal Medicine at http://www.annals.org/, and Epidemiology at http://www.epidem.com/). Information on the STROBE Initiative is available at www.strobe-statement.org.
