# Supplementary material for: Prevalence and predictors of secondary traumatic stress symptoms in health care professionals working with trauma victims: A cross-sectional study
Source: PLoS One. 2021 Feb 23;16(2):e0247596. doi: 10.1371/journal.pone.0247596 (PMC7901735; doi:10.1371/journal.pone.0247596)
Supplement: Attachment — Submitted filename: Response to Reviewers.docx [file pone.0247596.s003.docx]

**Editor:**

*Thank You so much for the suggestions.*

1) As the reviewer pointed out, the authors should explain the results of correlation analysis as briefly as possible.

*As the Editor suggested, it was explained as briefly as possible.*

2) I would advise using italics for letters used as statistical symbols or algebraic variables, such as M, SD, n, R^2^, B, t, p, r, N, BE, F, f^2^.

*As the Editor suggested, the edit was done.*

3) If there is no study or few studies of nurses in Poland which shows the relations between support from their friends and family and STS symptoms for now, the authors had better mention it after the sentence in L257.

*As the Editor suggested, the edit was done.*

4) The authors should add the reliability (Cronbach’s alpha) of the Job Satisfaction Scale.

*As the Editor suggested, the reliability (Cronbach’s alpha) of the Job Satisfaction Scale was added.*

5) I’d like to suggest that the authors move the sentence in L388 (A version adjusted for…) to before the sentence in L379 (The tool consists of …).

*As the Editor suggested, the edit was done.*

6) In Table 3, the beta value of job satisfaction for avoidance is -0.225. Is this correct? If so, denial should be placed above job satisfaction.

*As the Editor suggested, it was corrected.*

Minor errors.

L41: Two positive cognitive strategies → Two cognitive strategies

*As the Editor suggested, the edit was done.*

L43: other analysed variables → other analyzed variables

*As the Editor suggested, the edit was done.*

L97: overtone that → overtone than

*As the Editor suggested, the edit was done.*

L368: range = 0 - 35 → range = 7 – 35

*As the Editor suggested, the edit was done.*

L384: -3 (I certainly disagree) → 0 (I certainly disagree)

*As the Editor suggested, the edit was done.*

L384: 3 (I certainly agree) → 6 (I certainly agree)

*As the Editor suggested, the edit was done.*

L444: r < 0.001 → p < 0.001

*As the Editor suggested, the edit was done.*

L459: variables explaining → variables explained

*As the Editor suggested, the edit was done.*

L493: denial (0.232) → denial (β = 0.232)

*As the Editor suggested, the edit was done.*

L587: pain and suffering what can → pain and suffering, which can

*As the Editor suggested, the edit was done.*

L636: developing own work scheduled → developing own work schedule

*As the Editor suggested, the edit was done.*

It seems preferable to revise as below:

L43: other analyzed variables, i.e., denial, workload….

*As the Editor suggested, the edit was done.*

L207: The negative connections of satisfaction from helping with STS symptoms, compassion fatigue, and burnout were presented in the study of American nurses.

*As the Editor suggested, the edit was done.*

L244: a significant role in alleviating STS symptoms.

*As the Editor suggested, the edit was done.*

L272: the occurrence of the negative relations of beliefs relating to both the goodness of the surrounding world and its comprehensibility to STS symptoms.

*As the Editor suggested, the edit was done.*

L419: t (417) = -1.336

*As the Editor suggested, the edit was done.*

L423: t (417) = -0.496

*As the Editor suggested, the edit was done.*

L426 (It would be better to rename several variables listed in Table 1 as follows): STS intrusion, STS avoidance, STS negative cognition and mood, STS arousal and reactivity, COPT positive cognitive restructuring, CPOT downward comparison, CPOT resolution/acceptance, CPOT denial, CPOT regret.

*As the Editor suggested, the edit was done.*

L580: The results of correlation analysis indicated that STS symptoms are….

*As the Editor suggested, the edit was done.*

L641: may favour the occurrence of secondary posttraumatic growth (SPTG).

*As the Editor suggested, the edit was done.*

L648: use not only social support from the close ones but also various forms of support….

*As the Editor suggested, the edit was done.*

L652: significant means for the reduction….

*As the Editor suggested, the edit was done.*

**Reviewers:**

Reviewer #1: Thank you for your appropriate revision. You have responded to all my comments appropriately. I wish you the best of luck in your research.

*Thank You so much.*

Reviewer #2: Dear Authors, thank you for the opportunity to read the revised version of this manuscript. The vast majority of suggestions have been included, thank you for that. This version is precise and clear. For further processing, I suggest again not to include a detailed description of the correlation analysis. In this case, nothing has changed compared to the original manuscript: the correlation analysis (excluding Table 1) accounts for almost half of the content of the Results section. However, this is a preliminary analysis, and the regression is the core one. Table 1 is self-explainable though.

*As the Reviewer suggested, the Results section regarding correlational analysis was explained as briefly as possible.*

I also noticed some inconsistencies with the references. In line 639 you refer to: Dragana et al. [77] but there is no such reference on the list (77. Stanković A, Nikolic M, Nikić D, Arandjelović M. Job satisfaction in health care workers. Acta Medica Medianae. 2008; 47:9-12).
*Thank You so much for this comment. It was a little mistake in the author’s name. It was corrected.*

I hope you find these suggestions useful for your valuable manuscript.

*Thank You for the suggestions. They were very useful.*
